# Metabolic Therapy with Deanna Protocol Supplementation Delays Disease Progression and Extends Survival in Amyotrophic Lateral Sclerosis (ALS) Mouse Model

**DOI:** 10.1371/journal.pone.0103526

**Published:** 2014-07-25

**Authors:** Csilla Ari, Angela M. Poff, Heather E. Held, Carol S. Landon, Craig R. Goldhagen, Nicholas Mavromates, Dominic P. D’Agostino

**Affiliations:** Department of Molecular Pharmacology and Physiology, Hyperbaric Biomedical Research Laboratory, Morsani College of Medicine, University of South Florida, Tampa, Florida, United States of America; University of Florida, United States of America

## Abstract

Amyotrophic Lateral Sclerosis (ALS), also known as Lou Gehrig’s disease, is a neurodegenerative disorder of motor neurons causing progressive muscle weakness, paralysis, and eventual death from respiratory failure. There is currently no cure or effective treatment for ALS. Besides motor neuron degeneration, ALS is associated with impaired energy metabolism, which is pathophysiologically linked to mitochondrial dysfunction and glutamate excitotoxicity. The Deanna Protocol (DP) is a metabolic therapy that has been reported to alleviate symptoms in patients with ALS. In this study we hypothesized that alternative fuels in the form of TCA cycle intermediates, specifically arginine-alpha-ketoglutarate (AAKG), the main ingredient of the DP, and the ketogenic diet (KD), would increase motor function and survival in a mouse model of ALS (SOD1-G93A). ALS mice were fed standard rodent diet (SD), KD, or either diets containing a metabolic therapy of the primary ingredients of the DP consisting of AAKG, gamma-aminobutyric acid, Coenzyme Q10, and medium chain triglyceride high in caprylic triglyceride. Assessment of ALS-like pathology was performed using a pre-defined criteria for neurological score, accelerated rotarod test, paw grip endurance test, and grip strength test. Blood glucose, blood beta-hydroxybutyrate, and body weight were also monitored. SD+DP-fed mice exhibited improved neurological score from age 116 to 136 days compared to control mice. KD-fed mice exhibited better motor performance on all motor function tests at 15 and 16 weeks of age compared to controls. SD+DP and KD+DP therapies significantly extended survival time of SOD1-G93A mice by 7.5% (p = 0.001) and 4.2% (p = 0.006), respectively. Sixty-three percent of mice in the KD+DP and 72.7% of the SD+DP group lived past 125 days, while only 9% of the control animals survived past that point. Targeting energy metabolism with metabolic therapy produces a therapeutic effect in ALS mice which may prolong survival and quality of life in ALS patients.

## Introduction

Amyotrophic Lateral Sclerosis (ALS) is the most common adult-onset motor neuron disease, with a lifetime risk of 1 in 2000 [1] and a worldwide incidence of 1–3 new cases per 100,000 individuals [2]. Symptoms of ALS include spasticity, hyperreflexia, generalized weakness, fasciculations, muscle atrophy, and paralysis resulting in impaired respiratory function [3], [4]. Ultimately, this leads to death within 3–5 years of onset, most commonly from respiratory failure [3].

The causes of ALS are poorly understood: only 10% of the cases are inherited (familial ALS, fALS), and only 20% of these cases have been definitively linked to mutations in the superoxide dismutase 1 (SOD1) gene [5], [6]. The majority of the cases are sporadic (sALS, [7]), and the causes are largely unknown. Regardless of the type of ALS, patients exhibit neuronal cell death, which may be caused by excess glutamate and oxidative stress-induced metabolic dysfunction [8], [9]. Pathological hallmarks of ALS include mitochondrial dysfunction, increased oxidative stress, glutamate excitotoxicity, [10], [11], proteinopathy, glutaminergic dysregulation, metabolic dysregulation, and motor neuron death [9]. These disrupted cellular functions represent discrete targets for therapies that may ameliorate disease progression.

Evidence suggests that the histopathological and biochemical hallmarks of ALS result from impaired energy metabolism [12]. Previous work reported by Zhao et al. [3] has shown that a ketogenic diet (KD) or caprylic triglyceride [4] stalls the impairment of motor function and reduces death of motor neurons in the spinal cord of transgenic ALS mice (SOD1-G93A) by restoring energy metabolism through ketone body utilization. These transgenic ALS mice express mutant forms of the human SOD1 gene and multiple copies of the wild type (wt) human SOD1 gene; therefore, this mouse model is frequently used for studying the progression and mechanism of ALS pathogenesis [7], [13]. Under normal conditions, glucose is the primary metabolic fuel for the cells. However, alternative fuels such as ketone bodies or TCA cycle intermediates can potentially bypass the rate-limiting steps associated with impaired neuronal glucose metabolism and restore mitochondrial ATP production. Indeed, metabolic therapies such as therapeutic ketosis have been shown to effectively treat or alleviate symptoms of neurological disorders associated with aberrant energy metabolism, such as epilepsy, even in the presence of a persistent molecular pathology [14], [15]. In addition, coenzyme Q [16], creatine [17] and other metabolic intermediates were found to be effective against neuronal damage induced by excitotoxicity and mitochondrial inhibition.

Anecdotal reports from ALS patients describe symptomatic improvement in motor control following administration of the supplement component of the Deanna Protocol (DP, personal communications, Data S1). The DP is comprised primarily of arginine alpha-ketoglutarate (AAKG), and includes a number of other agents that were reported to preserve metabolic function and prevent glutamate excitotoxicity. The anecdotal reports in patients and existing data in animal models [3], [4] support the investigation of metabolic therapy for the management and treatment of ALS.

Based on previous research linking ALS pathology to metabolic dysfunction and anecdotal reports from patients, we hypothesized that supplying anaplerotic substrates and cofactors that are the primary ingredients of the DP, alone or in combination with a KD, would help to preserve metabolic function, alleviate ALS symptoms, and extend survival in an animal model of ALS. Our goal was to reverse or slow disease progression by supporting mitochondrial function and supplying alternative energy sources for neurons and glial cells.

## Materials and Methods

All animal procedures were performed within strict adherence to the NIH Guide for the Care and Use of Laboratory and Animals and were approved by the USF Institutional Animal Care and Use Committee.

### Experimental Animals and Diets

Fourteen B6SJL-Tg (SOD1-G93A) mutant transgenic mice (stock *#*002726) were purchased from the Jackson Laboratory (Bar Harbor, ME) at 60 days of age to test the palatability of the diets (SD n = 2; SD+DP, KD, KD+DP n = 4/group). Food consumption and body weight were monitored three times a week to ensure that the mice in each diet group consumed the same amount food and gained weight similarly. The data from these animals are not included in the results, and only functioned to allow optimal food composition for ensuing experiments.

**Figure 1:**
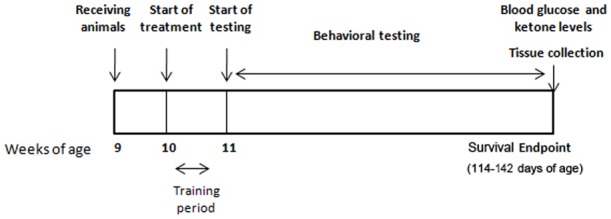
Experimental design for the study.

For the experiments testing the effects of diet on disease progression, forty-eight male B6SJL-Tg (SOD1-G93A) mutant transgenic mice (stock *#*002726) were purchased from the Jackson Laboratory (Bar Harbor, ME) at 60 days of age. Sex can affect the survival in this mouse model of ALS; therefore, only males were used during this study. All mice were maintained at a temperature of 21±1C with a relative humidity of 55±10% and 12 h of light. Figure 1 shows the experimental design. Animals were received at 9 weeks of age, while treatment and a one week training period began at 10 weeks of age. During the training period neuromuscular function tests were performed daily to allow familiarization. The experimental period started at 11 weeks of age from when tests were performed weekly until animals were euthanized.

The supplement used as part of the metabolic therapy was named Deanna Protocol (DP) and is described in Table 1. Animals were allowed to acclimate for seven days before any interventions. When they were 10 weeks old, they were randomly assigned to one of four groups: Standard Diet (SD; Control, n = 13); SD+DP (n = 12); Ketogenic Diet (KD, n = 11); or KD+DP (n = 12). At this time, neuromuscular training began. Mice in the SD group were fed standard rodent chow which is a low fat and high carbohydrate diet (2018 Teklad Global 18% Protein Rodent Diet, Harlan). Animals in the KD group received a custom-designed diet with a macronutrient profile similar to the clinically prescribed KD. Table 2 describes the macronutrient information of each diet. In the DP-treated groups, the SD and KD diets were mixed with DP at 22% by weight. DP was composed of AAKG (arginine alpha-ketoglutarate in 11 ratio, Primaforce, 10%; this dose has been confirmed to elevate serum level of AKG by metabolomics [18]), a GABA analogue that readily crosses the blood-brain barrier (Phenibut or beta-phenyl-gamma-aminobutyric acid HCl, Primaforce, 1%), bio-enhanced ubiquinone (HQ2, Tishcon, 0.1%), and medium chain triglyceride (MCT) high in caprylic acid (MCT oil, Life enhancement, 10%). All diets were mixed 11 with ddH_2_O to form a paste with similar consistencies. Diets were continuously replaced every other day to maintain freshness. Food and water were provided *ad libitum*. Once animals developed substantial motor impairments, food and a water-gelatin mixture were placed on the cage bottom to ensure free access. Body weight and food intake were monitored weekly. Since the different appearance of each diet made the groups visibly distinguishable, the experimenters were not blinded during the study.

**Table 1:** The macronutrient information of each diet.

Macronutrient Information	Standard Diet	Ketogenic Diet (KD)
% Cal from Fat	18.0	77.1
% Cal from Protein	24.0	22.4
% Cal from Carbohydrate	58.0	0.5
Caloric Density	3.1 Kcal/g	4.7 Kcal/g

**Table 2:** The composition of the metabolic therapy used in this study.

Deanna Protocol (DT)	% Composition in Diet
Arginine alpha-ketoglutarate (11 ratio)	10
Gamma-aminobutyric acid (GABA)	1
Ubiquinol (soluble CoQ10)	0.1
Medium Chain Triglyceride (MCT oil)	10

### Assessment of Motor Function

Motor function was assessed weekly using the accelerating rotarod test, grip test and hanging wire test. Prior to testing, mice received 5 days of training for motor function tests performance. During the study, performance on all three tests was monitored once weekly beginning at 11 weeks of age until they could no longer perform the test. To avoid any confounding effects of fatigue, the rotarod and grip strength tests were performed on one day, and the hanging wire test was performed three days later.

### Accelerating Rotarod Test

Mice were placed on an accelerating rotarod (AccuRotor Rotarod, AccuScan Instruments Inc.), which accelerated from 0 RPM to 40 RPM over 180 sec. The time maintained on the rod by each mouse was then recorded. Three attempts were given to each mouse on each testing day, and the longest duration was used for data analysis. Only mice that stayed on the accelerating rod for a minimum of 50 seconds at least once during the experimental period (week 11 or later) were included in the results. Due to disease progression, animals often exhibited severe muscle weakness and impaired coordination, at which time they were removed from the motor performance study. Following exclusion criteria, the cohort size for each group were as follows: SD, n = 12; SD+DP, n = 11; KD, n = 9, KD+DP, n = 9.

### Grip Test

Strength was tested using a four limb grip test (Grip Strength Test, Bioseb). The mouse was placed on a wire grid attached to the grip test box and allowed to grab the grid with all four paws before being gently pulled until it released its grip. The maximum force generated was recorded in grams. Each mouse performed the test three times. The highest value was recorded and used for data analysis. Following exclusion criteria, the cohort size for each group were as follows: SD, n = 13; SD+DP, n = 12; KD, n = 11; KD+DP, n = 12.

### Hanging Wire Test

Muscle endurance was assessed by the Paw Grip Endurance (PaGE) Test. Mice were placed on a wire lid and gently shaken to prompt the mouse to grip the grid. The lid was turned upside down over a housing cage and the time (sec) until the mouse released the wire lid was recorded. Each mouse was given three attempts to reach a maximum duration of 90 sec, and the longest duration was recorded. Only mice that reached 90 seconds at least once during the experimental period (week 11 or later) were included in the results. Following exclusion criteria, the cohort size for each group were as follows: SD, n = 9; SD+DP, n = 8; KD, n = 8; KD+DP, n = 8.

### Neurological score

Neurological score was determined twice a week until 100 days of age according to the criteria described in Table 3. Since the disease progression accelerates around 100 days of age the neurological score was assessed every day after 100 days of age (except days 119, 123 and 124). Mice that were euthanized are included in the category “4” on the subsequent days for analysis.

**Table 3:** Criteria of the neurological score.

0	Hind legs are fully extended during tail elevation
1	Hind legs are not fully extended during tail elevation
1.5	Abnormal gait
2	Toes curl under while walking
2.5	Difficulty walking while still using all four legs
3	Pulling hind legs behind their body, but still able to move them
3.5	Single hind leg paralysis
4	Complete hind limb paralysis and/or unable to return to upright position within 10 sec

Motor functions were evaluated by the same investigator throughout the study, and tests were performed in an environment with minimal stimuli such as noise, movement, or changes in light or temperature.

### Survival End-Point

Survival endpoint criteria were defined as meeting one of the following conditions: unable to return to the normal, upright position within 10 s following push over, or paralysis of hind limbs. Animals with survival time 2 or more standard deviations outside the mean were excluded from the survival curve. When the mice reached the endpoint, body weight was recorded and mice were humanely euthanized by IP injection of SomnaSol (50 mg/kg, Butler Schein, NDC #11695-4829-1.) Blood was collected by tail snip, and blood glucose and βHB were measured with the Precision Xtra Blood Glucose & Ketone Monitoring System (Abbott Inc.). Organs and tissue samples (Table 4) were harvested immediately, and their weights were recorded.

**Table 4:** The mean weight of organs and tissue samples collected from each group.

	SD	SD+DP	KD	KD+DP
**Ketone (mmol/ml)**	0.481±0.015	0.458±0.03	0.737±0.057	0.733±0.021
**Glucose (mg/dl)**	146.272±2.09	143.416±4.914	155.625±4.747	138±2.781
**Body weight (g)**	20.263±0.169	20.091±0.192	21.062±0.219	20.008±0.202
**Brain (mg)**	429±3	433±3	403±4	472±21
**Liver (mg)**	928±8	919±16	1194±33	1076±12
**Heart (mg)**	172±18	114±1	118±3	116±1
**Kidney (mg)**	351±3	361±4	358±4	374±3
**Spleen (mg)**	67±1	62±2	178±42	104±5
**Lungs (mg)**	152±2	149±2	141±4	162±5
**Gastrocnemius (mg)**	159±3	138±4	177±2	157±4
**EDL (mg)**	129±18	112±14	78±1	96±2
**Brown adipose (mg)**	133±19	55±1	42±5	46±1
**White adipose (V) (mg)**	22±6	20±3	13±5	12±1
**White adipose (S) (mg)**	29±3	26±3	26±2	12±2

All data is presented as mean ± SEM. (V: visceral, S: subcutaneous).

### Statistical analysis

Data analysis was performed using GraphPad PRISM version 6.0a. Significance between groups at each time point was determined by one-way ANOVA with Tukey’s multiple comparisons test. All data are presented as the mean ± standard error of the mean (SEM). Results were considered significant when p<0.05.

## Results

### Effect of treatment on blood glucose, ketones, organ weight, and bodyweight

**Figure 2:**
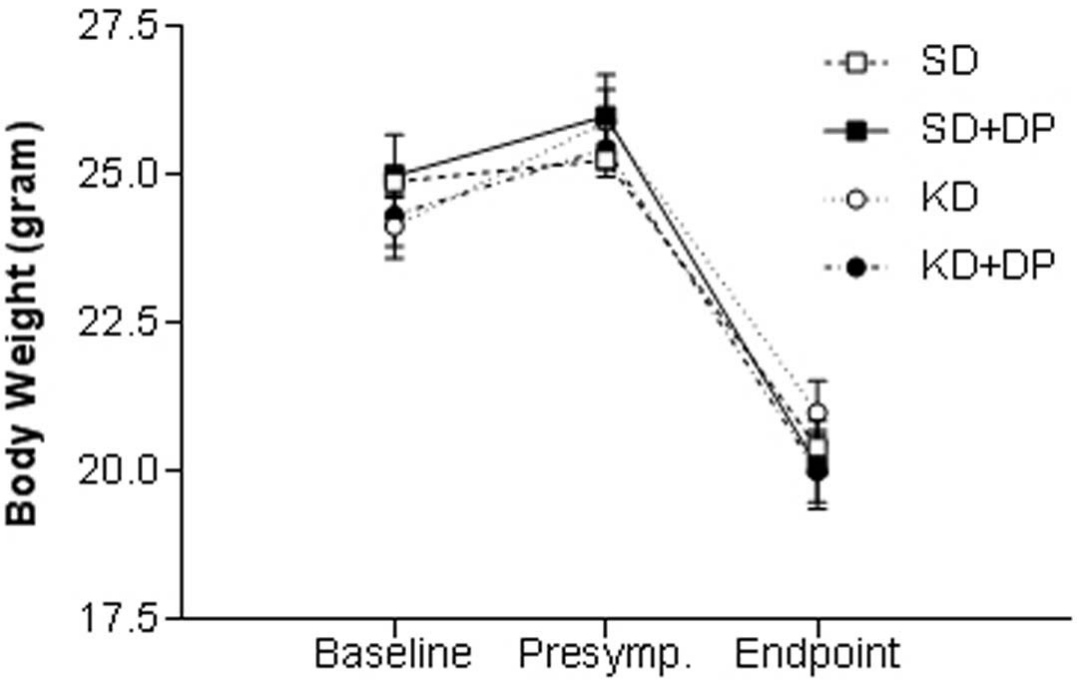
Effect of treatment on body weight in SOD1-G93A mice. There was no significant difference in body weight of SOD1-G93A mice between the different diet groups either before treatment started (Baseline), at presymptomatic stage (before neurological score 1) and at endpoint of the study. All data are mean ± SEM.

Blood glucose was not significantly different between the diet groups, while the blood βHB was 53% higher in KD and 52% higher in KD+DP compared to SD (Table 4). Brown adipose tissue was 30%, 38% and 46% greater in KD, KD+DP or SD+DP group, respectively, than in SD group (Table 4), though the body weight was not different between groups (Fig. 2, Data S2).

### KD and SD+DP improved motor function on accelerating rotarod, grip test, and PaGE test

**Figure 3:**
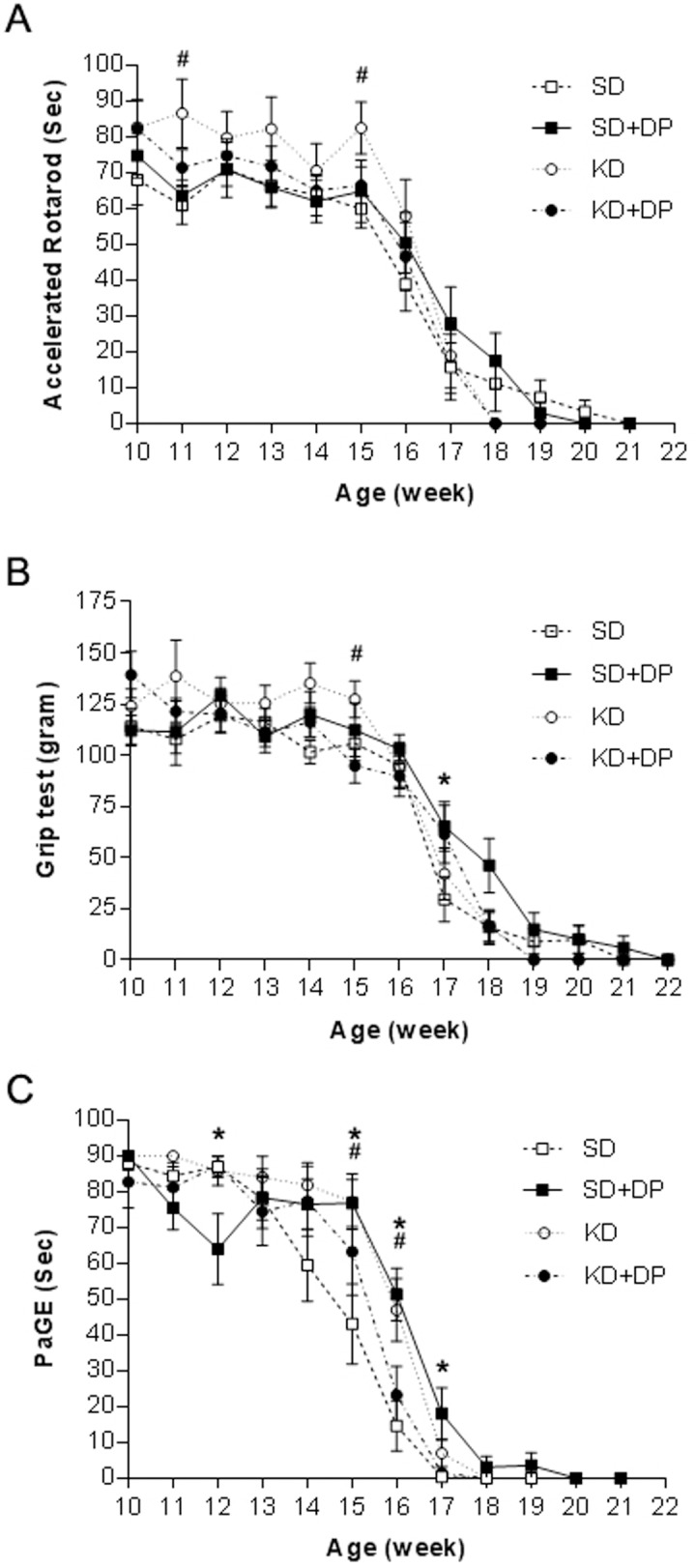
Improved motor function was observed in KD and SD+DP groups. A) KD group had significantly better result on accelerating rotarod during week 11 and 15 (p = 0.03, p = 0.018, respectively) when compared to the control group. (SD, n = 12; SD+DP, n = 11; KD, n = 9; KDDP, n = 9). B) Grip test showed better motor performance in KD group at week 15 (p = 0.015), while the motor function was significantly better at week 17 in SD+DP group (p = 0.039), compared to control. (SD, n = 13; SD+DP, n = 12; KD, n = 11; KDDP, n = 12). C) PaGE test showed better motor performance in KD group on week 15 and 16 (p = 0.027, p = 0.01), while the motor function was significantly better at week 12, 15, 16 and 17 in SD+DP group (p = 0.033, p = 0.023, p = 0.002, p = 0.019, respectively), when compared to SD group. All data are mean ± SEM. (SD, n = 9; SD+DP, n = 8; KD, n = 8; KDDP, n = 8). (*shows significant difference between SD and SD+DP groups; # shows significant difference between SD and KD+DP groups).

Accelerating rotarod data showed that KD mice had significantly better motor function during week 11 and 15 (p = 0.03, p = 0.018, respectively) compared to the control group (Fig. 3A, Data S1). Grip test showed better motor performance in KD group at week 15 (p = 0.015), while the motor function was significantly better at week 17 in SD+DP group (p = 0.039), compared to control (Fig. 3B, Data S1). PaGE test showed similar results. Better motor performance was recorded in KD group on week 15 and 16 (p = 0.027, p = 0.01), while the motor function was significantly better at week 12, 15, 16, and 17 in SD+DP group (p = 0.033, p = 0.023, p = 0.002, p = 0.019, respectively), when compared to SD group (Fig. 3C, Data S2).

### DP metabolic supplement delays disease progression and extends survival in the SOD1-G93A mouse model

**Figure 4:**
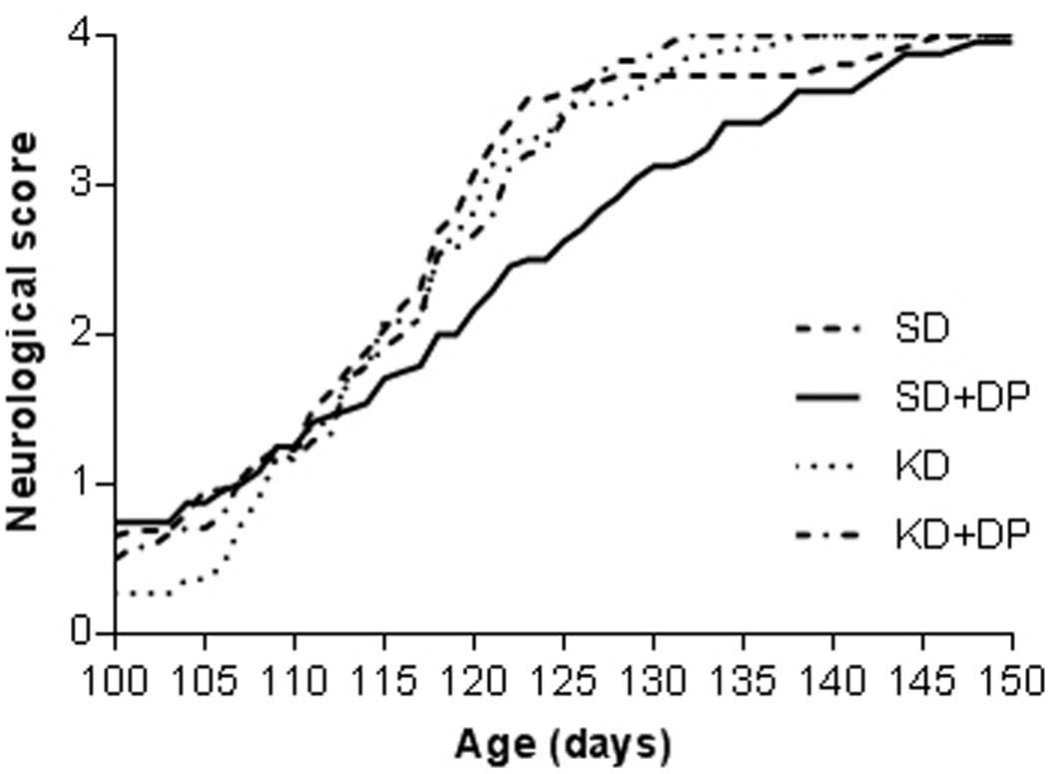
SD+DP delayed disease progression in SOD1-G93 mice. The difference in neurological score was significant between the control group (SD) and KD group on day 105 and 106, and the SD+DP group between day 118 and day 128 (except no data on day 119, 123, 124) implicating delayed disease progression in SD+DP group (p <0.05). All data are mean ± SEM.

Mice in the SD-DP group exhibited significantly improved (lower) neurological scores between days 118 and 136 compared to all other diet groups, indicating that progression of disease was significantly delayed (Fig. 4, Data S2).

**Figure 5:**
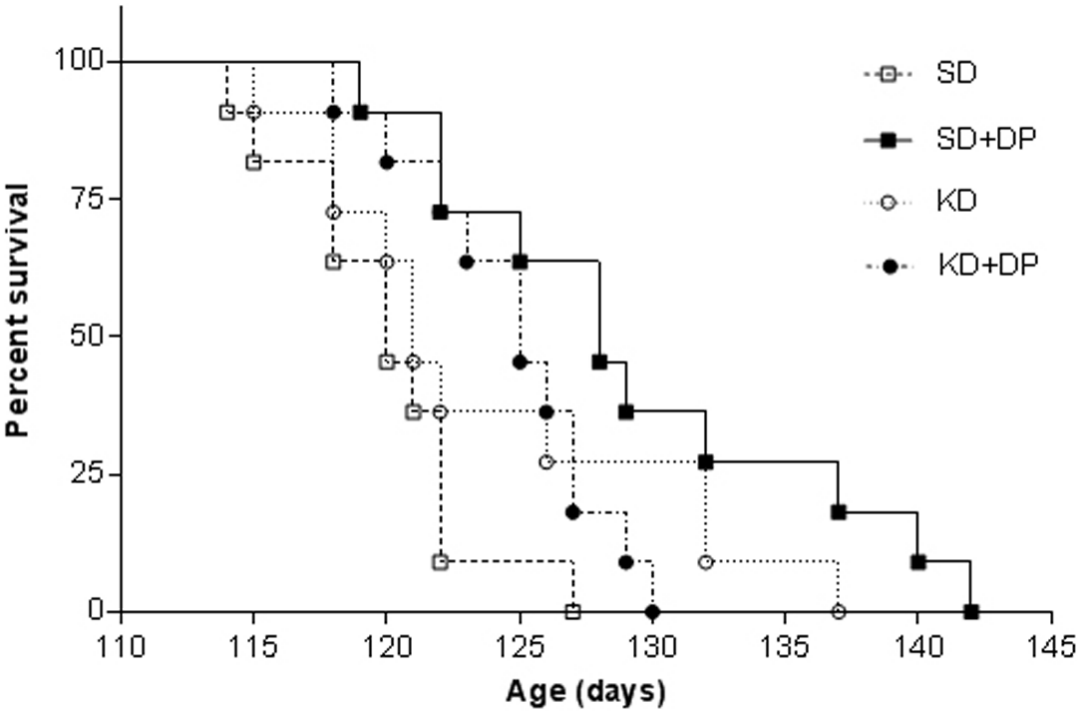
SD+DP increased survival time in SOD1-G93 mice. Kaplan-Meier survival plot of study groups. Animals receiving SD+DP showed significantly longer survival compared to control animals (p = 0.001).

Although the mean survival of SOD1-G93A animals was longer in all three treatment groups than the control group, this difference reached statistical significance only in the KD+DP (4.2%, p = 0.006) and SD+DP groups (7.5%, p = 0.001, Fig. 5, Table 5, Data S2). In fact, 63.3% and 72.7% of animals lived past 125 days in the KD+DP and SD+DP groups, respectively, while only 9% of the control animals did.

**Table 5:** The mean age at endpoint, increase in lifespan and the number of animals survived past 125 days are shown for each group.

Groups (n = 11)	Age at endpoint (days; mean ± SEM)	Percent increase in lifespan	% of animals survived past 125 days
SD	120±0.36		9
SD+DP	129±0.76	7.5% (p = 0.001)	73
KD	124±0.7	3.3% (p = 0.116)	36
KD+DP	125±0.37	4.2% (p = 0.006)	64

While all treatment groups had longer mean survival than the control group; the increase of survival time reached significance only at SD+DP group (7.5%, p = 0.001) and KD+DP group (4.2%, p = 0.006) compared to control. Animals in SD+DP and KD+DP groups had much bigger chance to survive over 125 days.

## Discussion

This study demonstrated that the Deanna Protocol (DP) supplementation added to a standard or ketogenic rodent diet improved motor function, delayed neurological deficits, and extended survival in SOD1-G93A mouse model of ALS. The major findings of the study show that during disease progression the SD+DP mice demonstrated improved motor function on rotarod, grip test, and PaGE test compared to control animals. Furthermore, the KD+DP and SD+DP groups exhibited longer survival times than the SD group.

### Mouse model

Reports vary regarding the onset of symptoms in this ALS model. Some studies report the onset of the symptoms at approximately day 90 [13] while other studies rather report day 60 [19], [20]. Hayworth [21] observed motor deficits during open field test from day 43 while Ligon [22] reported decreased grip strength at day 50. At 56 days, treadmill and inclined plane deficits have been noted [23], [24], while motor deficits detected were reported at day 60 by Mancuso [25]. Based on rotarod performance, our study showed that motor function started to decline around day 84; however several mice exhibited neurological signs as early as day 72. In summary, we observed neurological and motor signs of disease progression along similar timelines to others using the SOD1-G93A ALS mouse model, so our results can reasonably be compared with those available in the literature.

### Motor function improvement

The central hypothesis of this study was that by supplying additional metabolic substrates to compromised cells and mitochondria, we could stall disease progression and improve both motor performance and survival time. The main ingredient of the DP is AAKG, which is a salt of the amino acid arginine and alpha-ketoglutaric acid. These components are believed to promote enhanced blood flow, greater muscle protein synthesis and improve muscle strength by delivering AKG, a TCA cycle intermediate. Medium-chain triglycerides (MCT) were also added to the DP supplement and may serve a similar function as the AAKG. Namely, since MCTs do not require carnitine for oxidation and easily pass through the mitochondrial membrane, they can readily be metabolized to produce acetyl-CoA (also oxidized by way of the TCA cycle) or further converted to the ketone bodies, β-hydroxybutyrate and acetoacetate. We speculate that incorporating these intermediates in the diet may have ameliorated some symptoms of ALS by directly providing energy substrates, and thus bypassing rate-limiting steps of glucose transport or metabolism (*e.g*. pyruvate dehydrogenase), which may have been compromised by the disease [26], [27]. This may salvage motor neurons and underlie the observed improvements in motor performance. This hypothesis is supported by the fact that nutritional ketosis with KD or MCTs have been shown to increase physical endurance in mouse models of ALS [3], [4] and are widely used in athletics to prolong endurance.

Anecdotal reports from ALS patients taking the Deanna Protocol supplement complex describe improvement in mobility, strength and gait or slowed progression of ALS-related motor impairments (personal communications). Mice in our study that were fed with KD or the DP supplement showed better motor performance similar to what was found in previous studies [3], [4]. From these results it is clear that elevated blood ketone levels enhances motor performance, but is not sufficient for extending survival in this model of ALS. Zhao et al [4] reported that caprylic triglyceride-fed mice showed about a 2.5 fold increase in the blood concentration of circulating ketones compared to animals on control diet, while in the present study we found only a moderate blood ketone level elevation compared to the control diet, which likely led to a less prominent motor function improvement in KD and KD+DP groups.

The Deanna Protocol also contains GABA, an inhibitory neurotransmitter that regulates neuronal excitability throughout the nervous system, and is directly responsible for the regulation of muscle tone [28]. In healthy animals, dietary GABA does not readily cross the blood-brain barrier BBB and may be broken down by digestive enzymes. In ALS, however, the BBB is leaky, which may allow GABA to cross directly into the brain from the circulation [29], [30], [31]. Furthermore, the GABA analogue used in this study is conjugated with a 6-carbon ring, which makes it lipid soluble and allows it to pass directly through the BBB [32]. Thus, it is likely that dietary GABA was able to directly affect neuronal excitability in this study. Reduced excitability may underlie the improved motor control observed in the SD+DP group, though it is not clear why the KD+DP group apparently did not benefit from its addition.

Ubiquinol was added to the DP supplement since it is an essential component of the electron transport chain in mitochondria, thereby supporting mitochondrial function and ATP production. Although the amount used was small relative to the other components, the dose was 200–300 mg/kg in mice eating an average of 4–6 g/day, which has been proven effective for enhancing exercise capacity and suppression of oxidative stress [33], [34]. MCTs have also been shown to increase physical endurance in humans and in mouse models of disease [35], [36], including Alzheimer’s [37] and ALS [4]. In recent years, MCT oil and coconut oil have come into popular use for people with Alzheimer’s disease, other dementias, Parkinson’s disease, ALS, and other neurodegenerative diseases in which there is impaired glucose metabolism in the affected areas of the brain and/or peripheral nervous system [4], [38], [39]. The goal of such treatments is to deliver alternate metabolic substrates that bypass the problem (impaired glucose transport and insulin resistance) and may restore metabolic function in glucose-starved cells.

### Extended survival

To date, the only therapy offered to ALS patients to extend survival is riluzole, which offers only a modest (2–3 months) extension of survival in some patients, and has considerable side effects [6]. Therefore, studies on ALS transgenic mice are crucial to test potential therapies that not only improve motor function, but extend survival, especially if anecdotal reports in humans suggest a therapeutic effect. Interestingly, although we observed an increase in survival for the SD+DP and the KD+DP groups, this effect has not been observed by others using therapies that target energy metabolism [3], [4]. These results support further research, since increased motor function adds to improved quality of life, and extension of survival time is a primary clinical goal for ALS patients.

### Primary site of toxicity

It is unclear whether the primary site of toxicity in the SOD1-G93A mice is in the skeletal muscle [40] or the motor neurons [41], but it is likely that the mutated toxic form of SOD1 is expressed in more tissue types [2]. Therefore, supporting the mitochondrial function of many cell types is a desirable therapeutic approach.

Recent studies have shown that even a complete rescue of motor neuron cell bodies does not cure mSOD1 mice [42], [43], [44] suggesting that preserving the normal function of motor neuron cells is therapeutically not sufficient, since the rescued motor neurons are unable to recreate destroyed neuromuscular junctions (NMJ) [2]. Other attempts that rescue only motor neurons have also failed to halt progression [45]. The primary site of mSOD1 toxicity is likely to be represented in several other cell types, such as glial cells (astrocytes, microglia, or Schwann cells) and muscle fibers [2]. Recent study shows that astrocytes expressing mSOD1 were able to trigger motor neuron death through a mechanism involving oxidative stress and NGF production [46]. Other studies showed similar results suggesting that the astrocyte could be a site of mSOD1 toxicity [47], [48]. Indeed, decreasing mSOD1 expression in astrocytes also delayed disease onset in mSOD1 mice [49]. Other studies highlight the importance of the interdependence between neurons and astroglial cells. A recent study shows improvement in motor function and survival after using dichloroacetate (DCA) to treat astrocytes from SOD1-G93A mice [50]. DCA is a well-characterized inhibitor of pyruvate dehydrogenase kinase (activating PDH), enhancing glucose and lactate oxidation to CO2, and reducing lactate release in astrocytes while having little or no effect on neurons [51]. DCA effectively restored mitochondrial function and phenotypical features associated with normal astrocytes, and improved survival and motor performance of the SOD1-G93A mice [50]. This study indicates that restoring the energy metabolism of astrocytes might be a promising therapeutic approach.

Currently, there is no agreement on whether the muscle fibers, motor neurons, or the astroglial cells are affected first by the disease. To maintain the function of the NMJ and the healthy contact between muscle fibers, motor neurons, and astroglial cells, mitochondrial support of all these cell types is needed. By providing an alternative energy source in the form of AAKG, the mitochondrial function of these cells may be preserved.

### Conclusions

Previous studies have reported that metabolic therapies in the form of the ketogenic diet [3] or caprylic triglyceride [4] enhance motor function and may attenuate neuronal dysfunction in a variety of diseases [52]. Our results support the idea that targeted metabolic therapies, like the DP, can improve motor function and survival time, though further research is needed to elucidate the cellular and molecular mechanisms underlying these results. Dose response studies, histological analysis, and metabolomic analysis will help to define the optimal formula to enhance metabolic substrate delivery and utilization to help mitigate the ALS pathology. The components of the DP supplement have a good safety profile and are readily available over the counter, which is an important consideration in moving this therapy into phase I clinical trial.

## Supporting Information

Data S1 **Testimonials of 26 ALS patients who report having success from using the Deanna Protocol.** Clinical data from PALS.
(DOCX)

Data S2 **Raw data of the presented results.**
(XLS)
