# Supplementary material for: Metabolic Therapy with Deanna Protocol Supplementation Delays Disease Progression and Extends Survival in Amyotrophic Lateral Sclerosis (ALS) Mouse Model
Source: PLoS One. 2014 Jul 25;9(7):e103526. doi: 10.1371/journal.pone.0103526 (PMC4111621; doi:10.1371/journal.pone.0103526)
Supplement: Data S1 — Testimonials of 26 ALS patients who report having success from using the Deanna Protocol. Clinical data from PALS. (DOCX) [file pone.0103526.s001.docx]

**Clinical Data from PALS:**

**Testimonials of 26 ALS patients who report having success from using the Deanna protocol:**

**Anthony Topazi – President Mississippi Power**

I had great trouble breathing and my neurologist measured my breathing to have a force vital capacity that was down to 84% of normal. She suggested I have a diaphragm stimulator installed in my chest. instead, I went on the Deanna Protocol, started exercising my lungs and two months later my breathing capacity improved to 94% of normal. Now that doesn’t happen for ALS patients. Muscles don’t get stronger.

**Deanna Tedone-Gage**

My father experimented with a number of supplements until we realized that taking three particular supplements (coconut oil, A-AKG and GABA) were reversing my disease. My coordination, speech, walking, and breathing improved. My twitching and tremors also lessened dramatically. Friends and family members noted my improvement and began to spread the word to others who had loved ones with ALS.

**MJBUK**

My answer is YES, I believe the Deanna Protocol has helped me and I am better than I would have been if I had not been on it.

**DBLTREE**

I started March 18th, 2013 and the Deanna Protocol has helped my swallowing, excess saliva, speech, balance and walking and I have maintained an ALSFRS of 43. I am thankful for the quality of life extension granted by God and the Deanna Protocol.

**Andrew**

Diagnosed with ALS in December 2011. Before doing the Deanna Protocol, I was in a steep decline for several months. I started the Deanna Protocol in December 2012. Immediately there was an improvement in breathing and muscle strength. For the approximately 6 months since, I have maintained my ALSFRS score. Thank you Deanna and Dr. Tedone. I have no doubt that I'd be stuck in bed by now without your Protocol.

**Andrew**

I started the Deanna Protocol and here are the results by month:

Walking with walker

Mo. 0 about 20 ft

Mo. 1 about 50 ft

Mo. 2 about 100 ft

Mo. 3 about 100 ft

Mo. 4 about 50 ft

Mo. 5 about 40 ft

Arm strength and hand grip measured in pulley fly repetitions

Mo. 1 28

Mo. 2 100

Mo. 3 200

Mo. 4 250

Mo. 5 300

Breathing measured by spirometer has been good and steady.

I had a temporary dip in months 4 and 5 in walking because I changed the A-AKG supplier which was a mistake. I have since gone back to NOW which seems to work best for me.

I think that getting the right amount of exercise is the key. Concentrate on enough cardio and stretching and very light strength and resistance. I think more frequency is better than long exhausting reps. A little bit at a time more often.

I was in a steep decline prior to starting Deanna Protocol. So steady is outstanding for 5 months. Also, I am taking 180 g of protein a day.

**Cathy**

Whenever I go too long between doses of A-AKG, I notice leg/foot cramps. I should be taking more A-AKG but I have only been taking 3 to 6 capsules a day. I have very few fasciculation’s since I've been on the Deanna Protocol.

**Ron**

Taken full Protocol for a month including 30grams of A-AKG. Cramps have almost completely gone away and twitching almost completely gone. My neurologist said everything has maintained since he last saw me in terms of hands, arms, legs, balance and strength.

**Griff**

I have not noticed an increase in progression since reaching the recommended levels of A-AKG.

**RC**

I am just getting started on the Deanna Protocol, I don't ever want to return to the ALS clinic because I don't care for their attitudes of “Oh we're sorry but there's nothing we can do, go home and die.” Shrug their shoulders and send you a bill. No thanks. And they basically scoffed at the Deanna Protocol. I hope in time the medical community will pay more attention to us and the Deanna Protocol.

**Daniel**

I like the Deanna Protocol: it gives me a sense that I am doing something proactive about my situation rather than sitting around watching it happen. And it is working.

My "brother” and I are having similar experiences: progression, while it is occurring, appears to be slow.

**JPSteeler**

I stopped rubbing coconut oil into my legs and arms a few weeks ago and I took a big step down on the mobility chart. I think I will start doing that again.

**Natalie**

There is no doubt that the A-AKG assists with the twitches. When I couldn’t get it for two weeks, I noticed severe increase in fasciculation’s. During those two weeks my energy stooped to an all-time low.

**IDYSLPR**

I am now on most of the supplements and I'm getting better...really. And the coconut oil I am taking with honey, a bit easier to get it down. I am up to 3 tablespoons a day, and am using it as massage oil every day.

**TERICRUTCH**

I just wanted you all to know that I was turned down by Forbes Norris to be in a clinical trial because I am too strong. I’ve been on the Deanna Protocol for about 11 weeks and I believe that following the Protocol is key to maintaining my strength and health. So thank you Deanna and Dr. Tedone - and God Bless you both!

**JOEVIP**

I really feel the Deanna Protocol is helping hold off the disease and I will continue to take it as long as I keep feeling its working.

**TINALOVESLIFE**

The Deanna Protocol has made me Stronger, Steadier, and More Energy...Oh My! I was diagnosed 9/11/12. Spiraling abilities and my inability to be able to tolerate Rilutek led me to search elsewhere. The Deanna Protocol sounded right and natural. Alternative medicine has historically worked well for me most of my life. Physical, occupational, and speech therapies weren't doing much to stave off the disease and I was getting profoundly weak and tired. I dumped those therapies in favor of water aerobics 3 times a week, began ramping up dosages as per the Protocol two weeks ago, attend massage therapy once a week (arthromassage and manual ligament therapy), and began eating for my blood type.

**JSINTINK**

We really feel that the Deanna Protocol is preventing the disease from progressing in my father. He has:

1. Much improved strength and stamina

2. Lower leg cramping has improved (Less Cramping)

3. Twitching has improved from occasional twitching to rarely twitching

4. No noticeable change in state (Which is a really good thing) in the following areas: Speech, Swallowing and Saliva.

**TEAMCRANNY**

I have been on the Deanna Protocol about 7 days and the twitching of my major muscles groups has pretty much disappeared. I recently ran out of A-AKG and the twitching returned full force within 12 hours of missing a dose. I have also noticed that I have not tripped on my foot (drop foot) since starting the Protocol. I also think that with the exercise and Protocol supplements including the Coconut Oil is helping slow the progression. I have also started Thai Yoga Massage, and I have improved flexibility and motion. I was stretching just yesterday, and my bicep appeared hard and shaped like a muscle. This hasn't been there for over a month. Flexing only resulted in flabby arms, and right now, I am feeling pretty good. Not lifting anything heavy, but again, instills hope!

**Annie**

The Deanna Protocol, which my mother religiously subscribes to (a plethora of GNC-available supplements) has shown to significantly reduce her muscle spasms/twitching, so we are PLEASED AS PUNCH to see that the decline is slower moving. Woohoo!

**Gangwall**

My wife is on Deanna Protocol and has advised she is taking only AAKG .Apart from that she daily massages virgin organic coconut oil on the atrophied muscles once a day.

She does not like/unable to take daily dosage of coconut oil

**Johanne**

My 81-year old father with ALS for two years has followed the Deanna Protocol for the past few months. There has been an improvement with regard to food. He can eat everything except red meat. He can now eat things like raw tomatoes and chicken. Before the Deanna Protocol, it was impossible to eat these foods. His swallowing has improved, he is no longer choking. His encouragement has been an improvement in his speech, he speaks better, he no longer swallows the words, his voice is clearer and less hoarse. His lung capacity is better, and he sleeps very well. At night, he has almost no mucus. Dr. Tedone, a big thank you for your commitment. My father is better, using the Deanna Protocol, he lives better. (Translated from French by Cornell Christianson.)

**Steven**

In January, my sister’s number was 30 and since then her functional numbers have been at 28. The muscle twitching has stopped. She was off the A-AKG for a short period in May when she had her feeding tube inserted. The twitching started again and then stopped when she began taking the A-AKG again. She has declined since she started the Deanna Protocol however the rate of decline may have been greater without it. Not having her muscle twitch is a blessing. She is very diligent about taking the most important items in the Protocol. She doesn’t take all of them anymore because she has to crush the pills and use the feeding tube to take them. She finds it easier if she crushes the pills, puts them in a small amount of water and lets it stand awhile, then uses the feeding tube. Thank you for efforts to defeat ALS. People just need hope.

**Claude**

In December, 2012, we saw the television coverage on CBN, with the bright face of Deanna and the news that Dr. Tedone had discovered a treatment for ALS. Although it seemed crazy that a solution exists in the world (the Deanna Protocol) and nobody knows about it, especially here in France, where my father is fighting for his life with the horrors of ALS. A friend from the United States brought us the A-AKG and finally we found the right amount for him to take, doses at all hours awake. Immediate effect: disappearance of cramps and almost total disappearance of the fasciculation’s. I wish to thank Dr. Tedone: extremely available and open to discussion. I hope to meet him one day "for real" and thank him in person. (Translated from French by Cornell Christianson.)

**Gilles**

My ALS was making me very tired and my physical condition was deteriorating rapidly. I was in a very dark tunnel. Then I began taking the products of the "Deanna Protocol" limiting myself to the most important. After three weeks, I no longer had big problems with swallowing and choking. After 5 weeks, I found myself standing without falling on the buttocks. After 6 weeks, I noticed with astonishment that I strum naturally with 6-7 fingers. Today, some gestures are more natural like putting on slippers, holding a glass or fork, etc. I feel more dynamic. In view of the severity of ALS and its impact on everyday life, it is imperative that the scientific and medical community be interested seriously to refine, verify, and disseminate to the largest number of voluntary patients without delay the Deanna Protocol. (Translated from French by Cornell Christianson.)

Garry I simply want to thank you for all the fine ALS related research to which you have committed yourself, as well as your willingness to share the results of it with people who suffer from this disease. Briefly, though I have been taking Rilutek for about a year and a half, I am on but my fourth week of the protocol. Although I possess no scientific measure to gauge results, in the very least, I believe the progression of the characteristic symptoms have been retarded. I also " feel” that I have regained maybe 5 to 10% of the strength in my left leg. Additionally, at times, I know that I regained certain sensations in that same leg – – such as being able to flex muscles or even sense minor pain that should be associated with the stiffness that I had been experiencing in that same limb. The only negative side effect that I have known – – and naturally, this could be due to the progression of disease itself – – is a slight dizziness when standing without support. But I admit to this happening generally when I am tired or lacking sleep.

**Gerry** Just for the record, I have been on the Deanna Protocol for eight consecutive weeks now. Today, for the first time in months, I feel comfortable enough to walk around the house without the use of a cane.

**Graphs of ALSFRS-R scores for ALS patients.**

Overview

Below are graphs for 41 individuals with ALS that have voluntarily submitted an ALSFRS score.

The graphs below follow these criteria –

- The “Onset of Symptom Date” is an ALSFRS Score of 48, and therefore the first score.
- The 40 graphs are of registered site users
- The 40 graphs have at least 4 data points to plot (ALSFRS Scores) in the WFND database (including the “Onset of Symptom Date”. Each of these points is represented by a “blue dot”.
- The “Patient ID” is at the top of the graph to maintain the patient’s anonymity (example: Graph #1 is Patient 13).
- The average decline in score for individuals with ALS who are not on the DP is 1 point per month. Any decline less than 1 point per month reveals the disease progress has been slowed.
- Patients on the DP were asked to periodically return to take the ALS FRS test. We do not know if they took enough AKG to stop muscle symptoms or enough GABA to stop spasticity.
- Since 6 PALS out of 40 patients had a score of 1 or higher, the success rate of the DP is 85%

Figure 1

Loss: 0.90/month

Figure 2

Loss: 0.20/month

Figure 3

Loss: 0.705/month

Figure 4

Loss: 0.61/month

Figure 5

Loss: 1.0/month

Figure 6

Loss: 0.7/month

Figure 7

Loss: 0.130/month

Figure 8

Loss: 0.555/month

Figure 9

Loss: 0.205 until 6/26/13 then loss of 39 points in 2 months until 8/18/13. Data point 42 represents

either a critical mass (the point at which the body has too much glutamate for the AKG in the DP

to protect the cells), or a point at which the individual with ALS stopped taking the DP.

Figure 10

Loss: 0.666/month

Figure 11

Loss: 0.79/month

Figure 12

Loss: 0.541/month

Figure 13

Loss: 0.127/month

Figure 14

Loss: 0.733/month

Figure 15

Loss: 0.888/month

Figure 16

Loss: 0.482/month; Data point 42 - possible beginning of critical mass or individual with ALS stopped taking the DP

Figure 17

Loss: 0.552/month

Figure 18

Loss: 0.038/month

Figure 19

Loss: 1.3/month

Figure 20

Loss: 0.2/month

Figure 21

Loss: 1.58/month

Figure 22

Loss: 0.368/month

Figure 23

Loss: 0.227/month

Figure 24

Loss: 1.16/month

Figure 25

Loss 1.0/month; Data point 44 – possible beginning of critical mass or individual with ALS stopped

taking the DP

Figure 26

Loss: 0.24/month

Figure 27

Loss: 0.517/month

Figure 28

Loss: 0.75/month

Figure 29

Loss: 0.42/month

Figure 30

Loss: 0.347/month

Figure 31

Loss: 0.24

Figure 32

Loss: 0.25/month

Figure 33

Loss: 0.375

Figure 34

Loss: 0.833/month
